# A study of nitrogen incorporation in pyramidal site-controlled quantum dots

**DOI:** 10.1186/1556-276X-6-567

**Published:** 2011-10-26

**Authors:** Gediminas Juska, Valeria Dimastrodonato, Lorenzo O Mereni, Agnieszka Gocalinska, Emanuele Pelucchi

**Affiliations:** 1 Tyndall National Institute, University College Cork, Lee Maltings, Cork, Ireland

**Keywords:** MOVPE, site-controlled quantum dots, dilute nitride semiconductors

## Abstract

We present the results of a study of nitrogen incorporation in metalorganic-vapour-phase epitaxy-grown site-controlled quantum dots (QDs). We report for the first time on a significant incorporation (approximately 0.3%), producing a noteworthy red shift (at least 50 meV) in some of our samples. Depending on the level of nitrogen incorporation/exposure, strong modifications of the optical features are found (variable distribution of the emission homogeneity, fine-structure splitting, few-particle effects). We discuss our results, especially in relation to a specific reproducible sample which has noticeable features: the usual pattern of the excitonic transitions is altered and the fine-structure splitting is suppressed to vanishing values. Distinctively, nitrogen incorporation can be achieved without detriment to the optical quality, as confirmed by narrow linewidths and photon correlation spectroscopy.

## Background

Quantum dots (QDs) are usually referred to as 'artificial atoms' due to the discrete nature of energetic structure and similarities in quantum properties. Application of individual QDs is envisioned in the fields of advanced optoelectronics, photonics, quantum information processing [1]. Among the properties required to be met are certainly precise control over positioning (e.g. inside a photonic cavity or waveguide), high optical quality (i.e. low or absent spectral meandering of the excitonic features) and high uniformity as a guarantee of technological scalability. In many respects, features like tailored symmetry properties (e.g. better than C_2v _for entangled photon emission), customised oscillator strengths, wavelength tunability and possibly many more, depending on the applications, are a necessity.

The system of pyramidal QDs is particularly versatile. It ensures a precise spatial control over a few nanometres. Extremely high spectral purity and uniformity has also been demonstrated [2-4]. Emission wavelength control of the system can be achieved by changing the composition and/or thickness of pseudomorphically grown epitaxial QD layer [4,5], the size and position of tetrahedral recesses [6], the size and distribution of QDs [7], and different excitonic transitions are accessible depending on the measuring geometry [8,9]. One possible alternative for tuning the emission wavelength (possibly closer to the one of the transparent optical transmission windows used in telecommunications) can be achieved with the help of dilute nitride materials. It's widely known that incorporation of nitrogen in small quantities has a huge impact on the band structure of (In)GaAs [10,11]. The most prominent modification is the shrinkage of the emission energy. Also, the incorporation of the small radius nitrogen atoms into InGaAs alloys grown on GaAs is expected to reduce the strain and thus enable the growth of layers that are thicker and/or have higher indium concentration.

In this study, we report the first outcomes of our investigations on growing diluted nitride quantum dots. Our study of the emission wavelength as a function of the flux of nitrogen precursor unsymmetrical dimethylhydrazine (U-DMHy) used during the metalorganic vapour phase epitaxy (MOVPE) growth process has demonstrated (for the first time in the site-controlled family) unambiguous shrinkage of the emission energy by at least 50 meV. Moreover, despite the typically reported degradation of optical properties in dilute nitride materials, we maintain relatively high quality in some of our samples, which is also proved by evidence of single-photon emission. We show that the exposure to the nitrogen precursor during the growth has a major impact on the optical properties, varying substantially excitonic linewidths, emission homogeneity and QD symmetry properties. We discuss our results comparing to a specific (reproducible) sample which demonstrated noticeable features [12]: the usual pattern of the excitonic transitions is altered and a fine-structure splitting (FSS) is suppressed to values smaller than the measurement resolution of 4 μeV, while until now, site-controlled pyramidal QDs have always demonstrated (with the only one exception [13]) a FSS - an indication of broken rotational symmetry. We performed for the first time photon correlation spectroscopy on nitrogen-containing single dots, confirming few-particle attributions.

## Methods

Pyramidal QDs were grown by low pressure MOVPE, with nitrogen as carrier gas [14,15]. The standard MOVPE precursors, namely trimethyl(-gallium, -indium, -aluminium), dimethylhydrazine (U-DMHy) and arsine (AsH_3_), were used. Growth temperatures quoted are thermocouple ones. An important parameter is the flux ratio U-DMHy/AsH_3 _which was altered during growths of different samples in order to track changes of QD optical properties. In Tables 1 and 2, summary of the growth conditions and optical properties for each quantum dot sample analysed is presented.

**Table 1:** Growth parameters and optical properties of the set A QDs

Sample	N-free	A1	A2	A3
*T*_G_, °C	730	730	730	730
AsH_3_/III	750	430	430	430
U-DMHy/AsH_3_	0	0.66	0.33	0.16
QD thickness, nm	0.5	0.5	0.5	0.5
Averaged QD PL, meV	1462	1454	1440	1427
FSS, μeV	13	<4	-	-

**Table 2:** Growth parameters and optical properties of the set B QDs

Sample	N-free	B1	B2	B3
*T*_G_, °C	655	655	655	655
AsH_3_/III	430	200	430	430
U-DMHy/AsH_3_	0	5.46	2.52	1.26
QD thickness, nm	1.2	1.2	1.2	1.2
Averaged QD PL, meV	1372	1321	1320	1333
FSS, μeV	-	-	≤90	16

**Figure 1:**
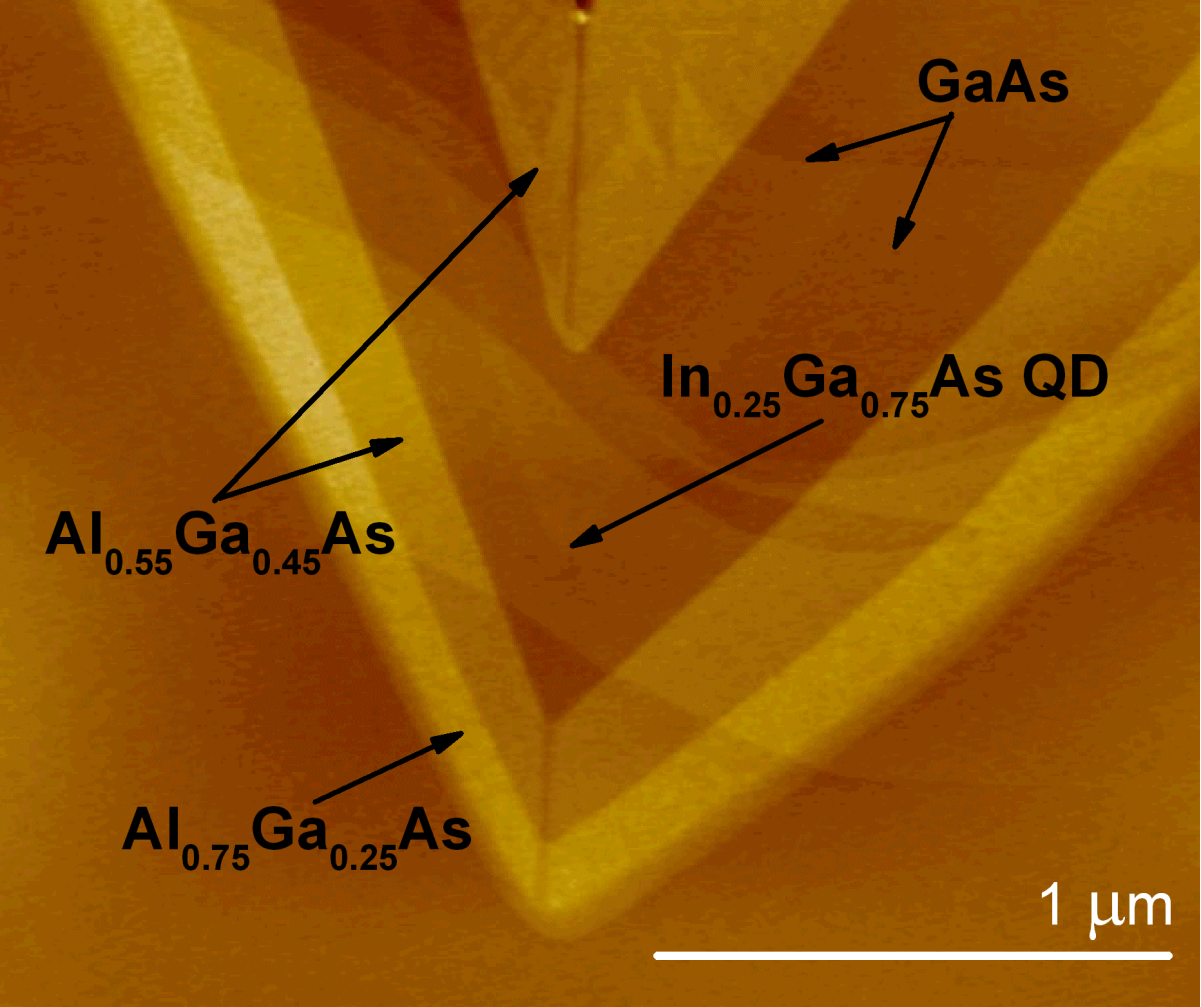
**The typical AFM cross-section image of epitaxial layers grown in a tetrahedral recess**. The layers are indicated in the image.

Pyramidal QDs were pseudomorphically grown on (111)B-oriented GaAs substrates. The substrate was pre-patterned with 7.5-μm pitch tetrahedrons by standard photolithography and wet chemical etching techniques. Thanks to capillarity and anisotropic decomposition effects, a single QD forms at the centre of each pyramidal recess [16]. The dot shape is determined simply by the self-limited profile of the underneath layers (GaAs in this case), their thickness and their composition. It must be said that a complex ensemble of quantum structures (three lateral quantum wires and a vertical one, three lateral quantum wells and three vertical ones) can form due to capillarity and capillarity induced segregation effects [17]. A typical sequence here used for the epitaxial layers can be identified in the representative cross-section atomic force microscopy (AFM) image in Figure 1. The first thick GaAs buffer layer is topped by a gradually variable composition Al*_x_*Ga_1−*x*_As (0.3 ≤ *x *≤ 0.75) layer and an etch stop film of Al_0.75_Ga_0.25_As which enables selective substrate removal during post-growth processing [15]. Here, to enhance the efficiency of pyramidal QDs, on some samples, a new advanced back-etching processing practice was applied, with a new gold bonding procedure replacing the usual wax application before substrate removal. Such apex-up geometry acts as a lens which helps to extract more light, enabling highly photon number sensitive measurements, such as correlations, efficient [18]. The active layer that forms the QD (In_0.25_Ga_0.75_As_1-*δ*_N*_δ_*) is embedded between outer Al_0.55_Ga_0.45_As and inner GaAs cladding layers. Two different sets of QDs have been grown: 0.5 nm (set A) and 1.2 nm (set B) nominal thickness at the temperatures of 730°C and 655°C, respectively. Results are also compared to non-nitrogen-containing dots.

The QD samples were characterised by micro-photoluminescence spectroscopy (microPL) at cryogenic temperatures. The core of the setup consists of a tuneable pulse repetition laser diode emitting at 633 nm, a helium closed-cycle cryostat and two spectrometers equipped with a charge-coupled device (CCD) or an array of InGaAs detectors. High-resolution, polarisation-resolved measurements of the FSS were carried out by placing a half-wave retardation plate and a linear polarizer in the optical axis of the system. Due to the presence of the FSS, the spectral positions of exciton and biexciton transitions typically follow counterphase sinusoids while changing polarisation angle. The value of the FSS is obtained by subtracting the corresponding biexciton-exciton positions. The resulting sinusoid amplitude gives the value of the FSS. The actual spectral resolution of approximately 18 μeV at 870 nm wavelength combined with a peak fitting procedure [19] enables a total resolution of approximately 4 μeV.

Photon correlation measurements were carried out in a typical Hanbury Brown and Twiss setup, equipped with silicon avalanche photo diodes (APD) with a low rate of dark counts (approximately 50 cps). Monochromators were used as narrow band-pass filters. Time-resolved features were measured with a single APD and a photon-counting card synchronised with an optical signal. The resolution of time-resolved features is determined by the instrument response function which can be very closely approximated by the Gaussian function with a width of 530 ps.

**Figure 2:**
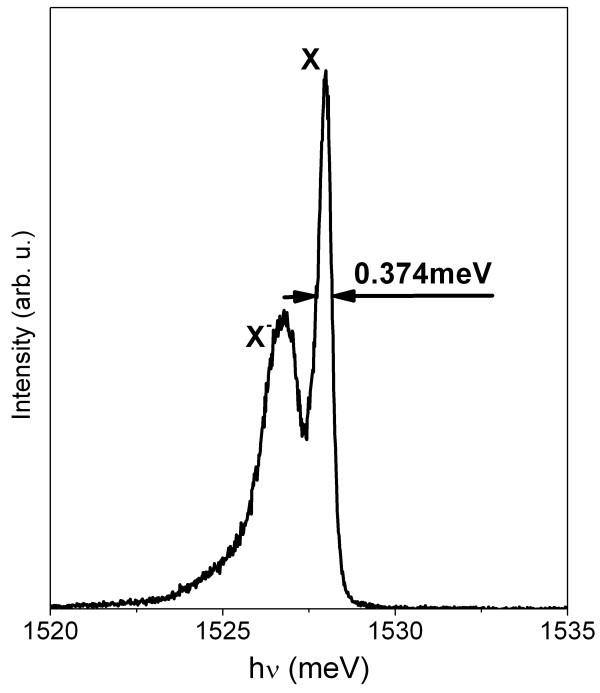
**The photoluminescence spectrum of a 15 nm GaAs/AlGaAs QW at 7 K**. Such narrow linewidths are a guarantee of low background impurities in our system

The purity of the epitaxial growth process was periodically tested by employing 15-nm-width GaAs quantum wells (QW) embedded between Al_0.3_Ga_0.7_As barriers [20,21]. The low-temperature photoluminescence from a QW is indeed sensitive to very low concentrations of unintentional impurities that are mostly unavoidable in MOVPE. Thus, a narrow linewidth of the excitonic transition is a reliable indicator of high reactor purity. The photoluminescence spectrum presented in Figure 2 from one of our QWs (the first reported to date by MOVPE with a free exciton linewidth below 400 μeV, see Ref. [20,21]) demonstrates a state-of-the-art linewidth. Such extremely high reactor purity is important as it provides favourable conditions for the growth of nanostructures of other type (e.g. our QDs).

## Results and discussion

**Figure 3:**
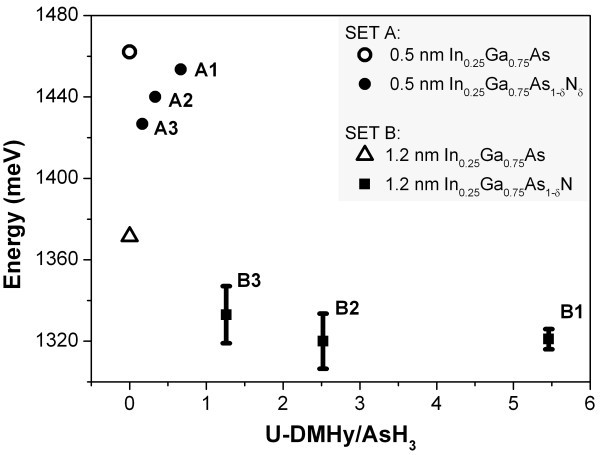
**Emission energy as a function of U-DMHy/AsH_3_**. Representative mostly red-shifted emission energy of In_0.25_Ga_0.75_As_1−*x*_N*_x _*single QDs as a function of U-DMHy/AsH_3 _flux ratio, compared with nitrogen-free QDs (open symbols).

Figure 3 shows part of the systematic study of the emission energy as a function of the U-DMHy/AsH_3 _ratio utilised during growth. Two different nitrogen-free In_0.25_Ga_0.75_As QDs samples [0.5- and 1.2-nm nominal thicknesses, growth temperature (thermocouple reading) 730°C and 655°C respectively, see also Tables 1 and 2] were chosen as reference points to count the emission energy shift. In general, no clear pattern can be found between the two ensembles, but lower temperature (ensemble B, 1.2-nm thickness QDs) gave us the strongest red-shift. In fact, the strongest QD photoluminescence red-shift was obtained of approximately 50 meV. A rough estimation based on the bandgap shrinkage measured experimentally in bulk dilute nitride In*_x_*Ga_1−*x*_As_1*-δ*_N*_δ _*alloys [22], suggests *δ *≈ 0.35%. We can give also an evaluation of the number of nitrogen atoms incorporated in the dot in this case, which can be obtained as follows: the base width of the dots grown at 655°C is around 50 nm, as it is the result of the self-limited value of the GaAs barriers. Assuming a dot thickness of approximately 3.5 nm (see Ref. [23]), a total of approximately 100,000 atoms in the dot can be calculated, from which assuming *δ *≈ 0.35% follows a few hundred nitrogen atoms as being incorporated.

Group B was grown at lower temperature (655°C) than group A, which is in general a more favourable condition to avoid nitrogen desorption from the surface [24]. Also, a higher flux ratio U-DMHy/AsH_3 _up to 5.46 was used compared to A samples, providing higher probability to trap nitrogen atoms. Nevertheless, a 35-meV energy shift has also been observed in the set A samples, even if the growth conditions were less favourable for nitrogen incorporation: a growth temperature of 730°C and the flux ratio up to 0.67. However, a good optical quality from QDs from the set A was only preserved in the sample with the smallest energy shift, while the other nitrogen containing dots showed broader linewidths, as high as 1 meV, and decreased intensity by more than one order of magnitude. Moreover, increasing U-DMHy in samples A did not trivially provide higher nitrogen incorporation. We do not have an explanation for this behaviour, and more work will be needed to clarify the exact incorporation dynamics in our samples.

**Figure 4:**
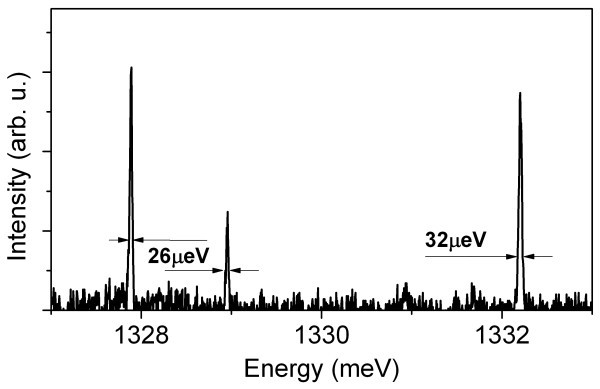
**Narrow linewidth transitions from the sample B2**. The linewidth values were obtained from Lorentzian fittings.

Nevertheless as a whole, we observed good optical quality, as shown in Figure 4, where a microPL spectrum from the sample B2 is reported. Extremely narrow lines (approximately 30 μeV) can be observed. We caution the reader that not all QDs in the sample emitted narrow lines, and unfortunately also dots with broad emission could be found.

Samples showing nitrogen incorporation do not show in general the uniformity of the emission properties (e.g. few milli-electron volt dispersion of emission energy, small dispersion of FSS, regular excitonic spectrum) that their counterpart without nitrogen demonstrated [2,3,25]. For example, a variety of few-particle effects have been observed and no constant pattern of excitonic transitions from the set B QDs has been measured, even if a significant part of them showed a biexciton emitting at lower energy (binding biexciton). It should be said, in fact, that non-uniformity should be expected in dilute nitride single QDs due to a high sensitivity of the energetic structure to small variations of the nitrogen incorporated (even a few tens of atoms correspond to a non negligible shift in emission energy and confinement energy). As a consequence, when nitrogen is significantly present, one can observe an increased non-homogeneity, which sometimes produces a free exciton emission energy distribution as broad as 30 meV (samples B2 and B3 in the Table 2).

**Figure 5:**
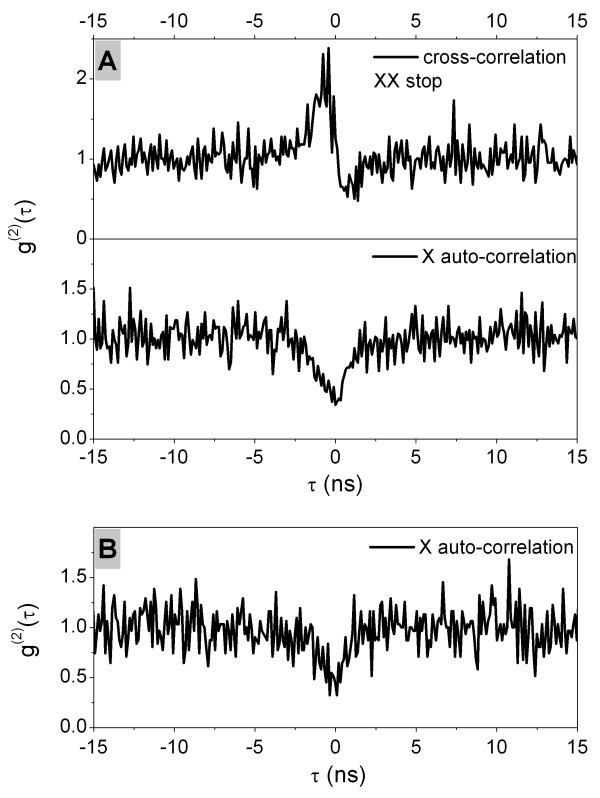
**Photon correlation measurements**. **(A) **Photon correlation measurements of excitonic transitions of QD from the sample A1. Biexciton photon detection events were used as stop signals in cross-correlation measurement. **(B) **Photon auto-correlation measurement of exciton transition of QD from the sample B2.

**Figure 6:**
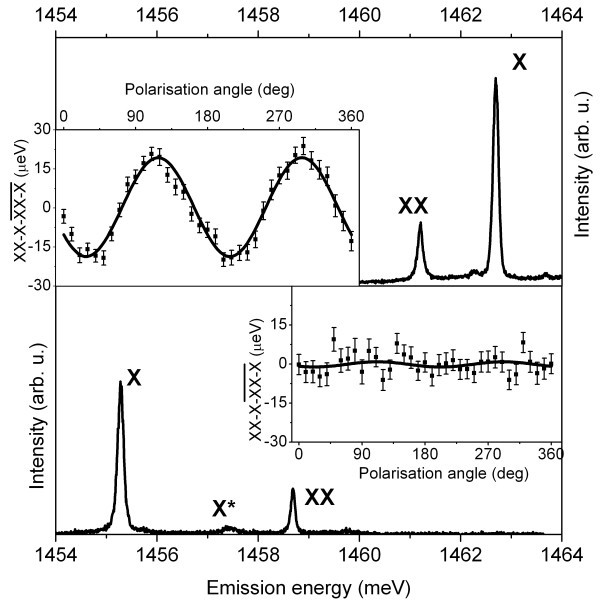
**PL spectra of pyramidal site-controlled QDs and fine-structure splitting measurement results**. (Top) In_0.25_Ga_0.75_As nitrogen-free 0.5-nm nominal thickness QD PL and (top inset) fine-structure splitting of 19-μeV demonstration. (Bottom) In_0.25_Ga_0.75_As_1−*δ*_N*_δ _*PL spectrum (note the antibinding biexciton) and (bottom inset) a typical fine-structure splitting measurement result, showing no detectable values.

The good optical quality found in some of our QDs allowed for photon correlation and single-photon emission testing. In Figure 5B, a clear dip in the second order correlation function *g*^(2)^(0) for the single exciton line (which goes to the value of 0.32) indicates non-classical light emission from the sample B2. Single-photon emission also was attested from the sample A1 (see Figure 5A). Photon bunching in the top curve obtained correlating single-photon detection events from two dominant transitions indicates biexciton-exciton recombination cascade (an increased probability to detect a second photon after recombination of biexciton). It's another strong proof of excitonic transitions type already assigned by time-resolved and excitation power dependent measurements in [12]. It should be noted that a third transition in the spectrum noted as X* in Figure 6 (main, bottom) was usually weak or absent, with a rare exception in a few QDs or at elevated temperature. Cross-correlation measurement between the exciton and this transition showed antibunching of photons (not shown). Taking into accounts the results of correlation and temperature dependent measurements, we identified this transition as a generic charged exciton.

When exciton and biexciton transitions could be identified in the samples from set A (A1, A3), negative biexciton binding energy (Δ*E*_XX _= *E*_X _− E_XX_) was always found to be a characteristic feature. We observe that even other QDs grown like the sample A1 but at lower temperature (700°C and 670°C, which we only discuss here in this contest), which reduces the width of self-limiting GaAs profile and thus the shape of a QD, exhibited a clear antibinding biexciton. The relevance of such modification is that generally the biexciton of counterpart QDs unexposed to U-DMHy is always binding (Δ*E*_XX _= 1.8 meV). A representative comparison of the spectra is presented in Figure 6, where the spectrum of 0.5 nm In_0.25 _Ga_0.75_As nitrogen-free QD is shown in the top part, while the typical spectrum of counterparts exposed to U-DMHy in the bottom. The relative position of biexciton transition (XX) in respect of exciton (X) indicates XX type (binding and antibinding, respectively).

In general, the change of excitonic pattern identifies a strong change of Coulomb interaction between photogenerated carriers within QD and possibly the change of geometrical properties [26]. However, the identification of such reasons in our case still requires further work so to obtain an elucidative theoretical model. It is nevertheless clear that the nature of the observed optical modifications cannot be attributed solely and simply to the nitrogen presence in the QD material, but more likely to its non-uniform distribution and/or incorporation, and its influence to the dot formation mechanism as a whole at given growth parameters. In fact, power dependency, time-resolved and fine-structure splitting measurements confirm that the biexciton of QDs in set B, where epitaxial conditions have been varied significantly, appears at lower energy (binding) than exciton, oppositely to the set A QDs.

As we reported elsewhere [12], we found that the sample A1 showed an unexpected welcome feature, a vanishingly small FSS, a result of interest as small but non-negligible FSS could be found in our nitrogen-free In*_x_*Ga_1−*x*_As QDs in GaAs barriers. For example, FSS of at least 7 μeV was always found in In_0.25 _Ga_0.75_As 0.5-nm nominal thickness QDs (grown at the same conditions as set A samples without exposure to U-DMHy). In the top inset of Figure 6 an example of FSS measurement of such nitrogen-free QD is presented - the sinusoid is directly related to the energetic position of exciton and biexciton and the amplitude of it gives the value of FSS.

**Figure 7:**
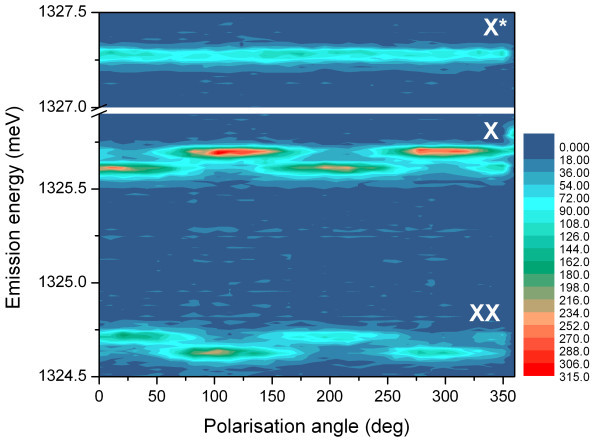
**Polarisation-dependent photoluminescence intensity mapping of the QD from the sample B2**. Due to exchange interaction and low rotational symmetry, two linearly polarised components are resolved in exciton (X) and biexciton (XX) transitions. Fine-structure splitting of 90 μeV can be resolved directly from the energetic distance between polarised components in each of the doublets. The third transition X* is identified as generic charged exciton.

As a matter of interest, we could not resolve FSS above our measurement resolution of 4 μeV in the sample A1. This result suggests that particular growth conditions could be exploited to grow QDs with improved rotational symmetry identified by very small FSS. To have better insight into this, we characterised all our samples and preliminary results of fine-structure splitting in set B QDs, when a clear exciton and biexciton transitions could be found, showed that a broad range of FSS values are present in our samples, and that despite being reproducible as such, A1 seems to be a unique example of vanishing FSS. In fact, although theoretical argumentations predict no FSS in QDs grown on (111) surface due to high symmetrical properties [27,28], values as high as 90 μeV could be found in the samples B3 and B2, an indication possibly of increased alloy disorder. An example of FSS presence in the sample B2 is shown in the Figure 7, where the QD photoluminescence intensity is mapped against polarisation detection angle. Exciton (X) and biexciton (XX) transitions are clearly identified as each of them is comprised of two linearly polarised and energetically distant components - the result of exchange interaction between electron and hole in a low rotational symmetry QD. The energy separation in both doublets of X and XX is equal to the FSS of 90 μeV. The third transition X* is attributed to generic charged exciton, as it does not show polarisation dependence due to the lack of exchange interaction [29]. The attributed nature of these transitions is consistent with excitation power dependent measurements (not presented), where exciton intensity rises linearly, while biexciton as a square of excitation power [30].

## Conclusions

In conclusion, we have presented the first broad study on nitrogen incorporation in single In_0.25 _Ga_0.75_As_1-*δ*_N*_δ _*site-controlled QDs. Nitrogen incorporation was attested by photoluminescence red-shift of at least 50 meV. Single-photon emission from selected QDs was proved by auto-correlation measurements. While nitrogen incorporation in thicker QDs grown at lower temperature resulted in a broad distribution of optical properties (emission energy and transitions linewidth non-homogeneity) and fine-structure splitting as large as 90 μeV, exposure to U-DMHy of thinner QDs grown in a specific set of epitaxial conditions (higher temperature) altered the excitonic pattern (an antibinding biexciton appeared) and fine-structure splitting values were suppressed below the setup resolution of 4 μeV.

As a consequence of our results, it is clear that the exposure of QD layer to U-DMHy during the growth could be exploited not only as an emission tuning mechanism preserving good quality of optical properties but also in a particular case to improve the rotational symmetry of pyramidal site-controlled QDs - a necessary feature for the efficient generation of entangled photons.

## Competing interests

The authors declare that they have no competing interests.

## Authors' contributions

GJ and LOM carried out optical characterisation of the samples and data analysis. VD and AG participated in the production of the samples, processing and microscopy characterization. EP conceived of the study, and participated in its design and coordination. All the authors participated in writing the draft, read and approved the final manuscript.
